# Supplementary material for: MetaRibo-Seq measures translation in microbiomes
Source: Nat Commun. 2020 Jun 29;11:3268. doi: 10.1038/s41467-020-17081-z (PMC7324362; doi:10.1038/s41467-020-17081-z)
Supplement: Supplementary file 10 — Supplementary Data 7 [file 41467_2020_17081_MOESM10_ESM.zip › File2/Confidence_High_Taxonomy/40905_out.krona.html]

Javascript must be enabled to view this page.

members
magnitude
magnitudeUnassigned
count
unassigned
taxon
rank

40905\_out

2

2
superkingdom
2

32066
phylum
1

203490
class
1

1
order
203491

1129771
1
family

32067
1
genus

species
1

SRS024504\_contig\_number\_contig-100\_5865.41715
157692

1239
1
phylum

1
class
186801

186802
1
order

31979
family
1

1485
genus
1


SRS047634\_contig\_number\_13868
319475
1
species
